# Supporting Treatment Adherence Readiness through Training (START) for patients with HIV on antiretroviral therapy: study protocol for a randomized controlled trial

**DOI:** 10.1186/s13063-016-1287-3

**Published:** 2016-03-24

**Authors:** Glenn J. Wagner, Sebastien Linnemayr, Bonnie Ghosh-Dastidar, Judith S. Currier, Risa Hoffman, Stefan Schneider

**Affiliations:** RAND Corporation, 1776 Main Street, Santa Monica, CA 90407 USA; Department of Medicine, UCLA, Los Angeles, CA USA; Long Beach Education and Research Consultants, Long Beach, CA USA

**Keywords:** HIV, Antiretroviral, Adherence, Readiness, Intervention, Randomized controlled trial

## Abstract

**Background:**

Few HIV antiretroviral adherence interventions target patients before they start treatment, assess adherence readiness to determine the timing of treatment initiation, or tailor the amount of adherence support. The Supporting Treatment Adherence Readiness through Training (START) intervention, based on the information-motivation-behavioral skills model of behavior change, is designed to address these gaps with the inclusion of (1) brief pill-taking practice trials for enhancing pretreatment adherence counseling and providing a behavioral criterion for determining adherence readiness and the timing of treatment initiation and (2) a performance-driven dose regulation mechanism to tailor the amount of counseling to the individual needs of the patient and conserve resources. The primary aim of this randomized controlled trial is to examine the effects of START on antiretroviral adherence and HIV virologic suppression.

**Methods/design:**

A sample of 240 patients will be randomized to receive START or usual care at one of two HIV clinics. Primary outcomes will be optimal dose-taking adherence (>85 % prescribed doses taken), as measured with electronic monitoring caps, and undetectable HIV viral load. Secondary outcomes will include dose-timing adherence (>85 % prescribed doses taken on time) and CD4 count. Primary endpoints will be month 6 (short-term effect) and month 24 (to test durability of effect), though electronic monitoring will be continuous and a fully battery of assessments will be administered every 6 months for 24 months.

**Discussion:**

If efficacious and cost-effective, START will provide clinicians with a model for assessing patient adherence readiness and helping patients to achieve and sustain readiness and optimal treatment benefits.

**Trial registration:**

ClinicalTrials.gov identifier NCT02329782. Registered on 22 December 2014.

**Electronic supplementary material:**

The online version of this article (doi:10.1186/s13063-016-1287-3) contains supplementary material, which is available to authorized users.

## Background

The success of HIV antiretroviral therapy (ART) remains dependent on high adherence [1–4]. While regimens have been simplified and are more forgiving, nonadherence is still a problem unsolved because many patients (40–70 %) have suboptimal adherence [5–7]. With the emergence of treatment as prevention [8] and patients starting ART earlier [9], often before having experienced any acute or advanced disease, the public health risks of nonadherence may be greater than ever. Studies suggest that patients with earlier-stage disease are less adherent to treatment [10, 11], and, if many patients are started on treatment before they are ready to adhere well, there is greater risk for an expanding community pool of resistance and less than adequate suppression of viral load and infectiousness to limit transmission. Treatment guidelines continue to emphasize the need for patients to be ready to adhere well before starting ART [9], but there are no established, reliable methods for determining pretreatment adherence readiness.

Most ART adherence interventions are designed to help patients achieve and maintain adherence readiness, but few target patients before they start treatment or tailor the amount of adherence support [12]. The Supporting Treatment Adherence Readiness through Training (START) intervention is based on the information-motivation-behavioral skills (IMB) model of behavior change [13]. It includes (1) brief pill-taking practice trials for enhancing pretreatment adherence counseling and providing a behavioral criterion for determining adherence readiness and the timing of treatment initiation and (2) a performance-driven dose regulation mechanism to tailor the amount of counseling to the individual needs of the patient and conserve resources. In a pilot randomized controlled trial [14], START had effects on both behavioral adherence and virologic suppression, unusual even for effective ART adherence interventions, which have typically improved one but not both of these outcomes [15]. The small sample size (*N* = 54) precluded adequate statistical power, but effect size estimates were 0.22 (dose-taking adherence), 0.75 (dose-timing adherence), and 0.41 (complete virologic suppression) over the first 6 months of treatment, with the latter two far exceeding the average 0.19 effect size found in a recent meta-analysis of ART adherence interventions that, like START, were not focused solely on patients already demonstrating adherence problems [12]. These promising results provide the rationale for the current, more rigorous, larger controlled trial of START.

In the present study, we are evaluating the effects of the START intervention on the primary outcomes of dose-taking adherence and undetectable viral load in a multisite randomized controlled trial, the protocol of which this article describes in detail. The primary objective of the study is to determine whether START is superior to usual care in helping patients achieve optimal (>85 %) dose-taking adherence, and complete viral suppression (undetectable HIV viral load) 6 months (short-term efficacy) and 24 months (long-term efficacy) after starting ART.

If effective, START will provide clinicians with an intervention that (1) informs providers and patients when the patient is ready to adhere well and start treatment, (2) enhances adherence readiness from the outset of treatment through the full course of therapy, and (3) tailors the amount of adherence support based on individual patient needs and performance, thus more efficiently using clinic resources, fostering better acceptance from providers and patients, and increasing the likelihood of successful program adoption and dissemination.

## Methods/design

### Study design

In this multisite randomized controlled trial, we will evaluate the effects of the START intervention on ART adherence and HIV virologic suppression. We will recruit 240 patients with HIV at two HIV clinics, with each randomized to receive either START or usual care at a 1:1 ratio at each clinic (60 patients in each arm at each site). Random assignment codes will be computer-generated and placed in a sequential list that will be maintained by the principal investigator. Assignments will be issued to study coordinators once candidates have enrolled and completed the baseline survey. The randomization will be stratified by prior ART history (naïve or experienced) and CD4 count (<350 vs. ≥350 cells/mm^3^) because of the influence that prior ART history may have on adherence and clinical outcomes, and also due to our interest in determining whether early-stage patients can adhere as well as other patients to treatment. There is no way to blind the participants to whether they are receiving the intervention; this could potentially influence adherence performance and retention, as clients may feel more or less incentive to perform well in light of whether they receive the intervention. We do not see a way to prevent this potential bias, nor do we have a way to distinguish such effects from actual intervention effects; however, this limitation will be cited in reports of study findings.

**Fig. 1:**
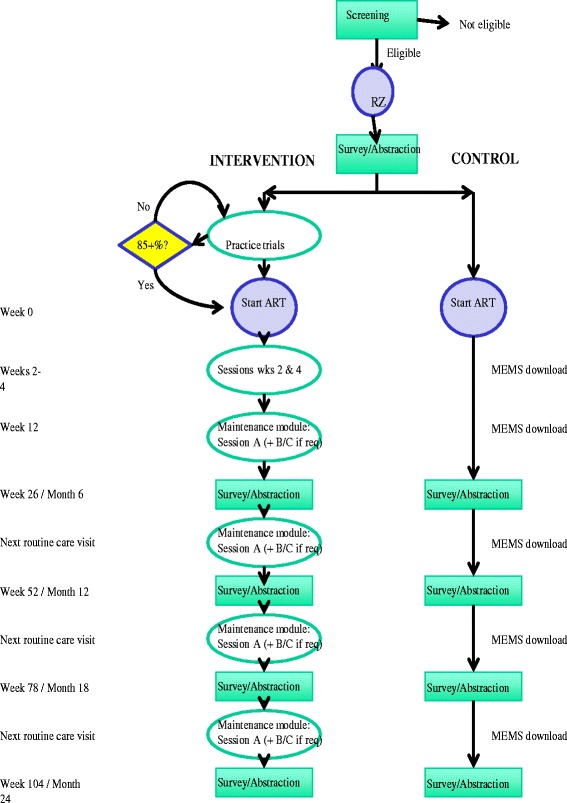
Flowchart of study protocol. *RZ* randomized, *ART* antiretroviral therapy, *MEMS* Medication Event Monitoring System

Primary outcomes will be optimal dose-taking adherence (>85 % prescribed doses taken), as measured with electronic monitoring caps, and undetectable HIV viral load. Secondary outcomes will include dose-timing adherence (>85 % prescribed doses taken on time) and CD4 count. Primary endpoints will be month 6 (short-term effect) and month 24 (to test durability of effect), though electronic monitoring will be continuous and a fully battery of assessments will be administered every 6 months for 24 months. See Fig. 1 for the flow of participant procedures through the protocol. Participants will be paid $30 for each completed assessment, as well as a $50 bonus if they complete all five primary assessments and $10 for each attended intervention session. To limit attrition, contact information will be collected at enrollment and verified at each follow-up visit. Furthermore, the study coordinator will check with all study participants at the midway point between surveys, which will be timed to coincide with regular scheduled clinic appointments.

The study protocol has been reviewed and approved by institutional review board (IRB) at the University of California, Los Angeles (UCLA), and a certificate of confidentiality has been obtained from the National Institute of Mental Health. Any protocol modifications will be submitted to the IRB for review, and participants will be informed if warranted. The trial is registered with the National Institutes of Health (NIH) ClinicalTrials.gov registry and assigned the number NCT02329782.

### Setting

The study will take place at St. Mary’s Comprehensive AIDS Resource and Education Clinic in Long Beach, CA, USA, and UCLA Clinical AIDS Research and Education (CARE) Center. The St. Mary’s clinic serves over 1500 clients with HIV, of whom about 60 % are nonwhite and 85 % are male. The UCLA CARE center has nearly 1300 clients with HIV, of whom 83 % are male and 40 % are nonwhite. Both clinics have highly productive research units that conduct primarily biomedical and therapeutics research. Both research units have highly trained study coordinators who are experienced in research ethics, evaluation procedures, protocol implementation, and provision of adherence treatment support and counseling (as part of research trials, not usual care), and they will be trained to be the project’s site coordinators and intervention counselors.

### Study sample

Eligibility criteria include (1) patient and provider plan to start or restart (not currently on ART) the patient on ART; (2) patient has stable health status and no acute opportunistic infections or medical conditions (including acute HIV infection) that warrant starting ART immediately, as determined by the patient’s provider; (3) detectable HIV viral load at last test (within past 6 months); (4) if CD4 count is <200 cells/mm^3^, patient is on prophylactic medication to limit risks associated with the intervention’s imposed delay in starting ART; (5) patient is age 18 years or older; and (6) patient speaks English. Providers will inform eligible patients of the study, and patients who interested in participating will be referred to the study coordinator for consent procedures, eligibility screening, baseline survey, and randomization. Written informed consent will be obtained from all participants at enrollment. For control patients, the baseline visit is scheduled for the same day or within 1 week of the screening assessment. The baseline visit for intervention patients will take place on the same day as the last pretreatment intervention session (once they achieve >85 % dose-taking adherence during a practice trial).

We calculated the size of effects that this sample can detect with 80 % power (two-tailed test) with regard to the primary outcomes. On the basis of the pilot study [14], we expect that 40 % of the control group will achieve optimal (>85 %) dose-taking adherence and 45 % will achieve undetectable viral load. With these expected rates in the control group, together with estimates of 10 % and 30 % attrition at months 6 and 24, respectively, our sample size of 240 will be powered to detect a 9 % difference between the two arms with regard to both optimal dose-taking adherence and undetectable viral load at both months 6 and 24.

### Adherence interventions

#### Usual clinical care

Usual care procedures to enhance adherence at the clinics will continue to be implemented for all participants. Strategies for assessing adherence readiness at the study sites include educating the patient about the importance of adherence, evaluating patient commitment and motivation to adhere to treatment, and assessing whether barriers to adherence need to be addressed before initiating treatment (e.g., unstable housing, substance abuse). Once ART is initiated, routine inquiries about adherence occur at regular follow-up visits (once or twice in the first month of treatment, and then every 3–6 months). What varies across individual patients is the staff member who discusses these issues with the patient (e.g., nurse, physician, case manager) and the amount of time spent on these issues. Structured, systematic counseling protocols and practice trials are not part of usual care. We will measure adherence support received by patients as part of usual care in the participant surveys, as described below.

#### START intervention

START is a comprehensive program of adherence training based on the IMB model of health behavior [13], which asserts that *information* about ART and the importance of adherence is a prerequisite but in itself is insufficient to alter behavior, and that *motivation* for adherence and *behavioral skills* to adhere well and overcome barriers are critical determinants of adherence. Motivation is fed by beliefs that the medication will be helpful (treatment efficacy) and confidence that one can adhere even in difficult circumstances (adherence self-efficacy). Behavioral skills such as problem-solving and adherence self-monitoring help to identify adherence barriers and effective solutions to these barriers. While not emphasized in the traditional IMB model, the social context in which adherence occurs and the support the patient receives can be important to all three of these components, both positively and negatively. One’s support network can be a source of information and can transmit beliefs about treatment that affect motivation. Social support can maintain or break down psychological well-being and psychosocial functioning, thereby impacting the patient’s ability to develop and use behavioral skills needed to adhere well. Each component of the IMB model is present in the START components and specific sessions, as shown in Table 1.

**Table 1:** Mapping of the START intervention’s conceptual framework and session content

Model component	Intervention component or mechanism	Sessions
Information	Provide information about HIV, ART, and the importance of adherence for treatment success and limiting resistance	Pre-ART session 1
Motivation	Discuss attitudes and beliefs regarding ART and adherence, and use MI to reframe and build positive attitudes	Pre-ART session 1, early ART week 2, maintenance phase
	Use of problem-solving and confidence rulers to improve adherence self-efficacy and sense of autonomy	Each session after the first one
Behavioral skills	Practice trials to practice adherence to planned regimen	Pre-ART sessions
	Review adherence results (self-management)	Each session after the first one
	Use problem-solving to identify adherence barriers and generate and evaluate solutions to these barriers	Each session after the first one
	Integrate regimen into daily routine	Pre-ART session 2
	Side effect management	Early ART and maintenance
Social support	Review source of social support (positive and negative) and discuss ways to enhance support for adherence	Pre-ART session 1, early ART week 2, maintenance phase

*ART* antiretroviral therapy, *MI* motivational interviewing, *START* Supporting Treatment Adherence Readiness through Training

START consists of pretreatment (including practice trials to determine readiness for and timing of ART initiation), early treatment, and ongoing maintenance training (using a performance-based dose regulation mechanism to tailor the amount and intensity) phases, each of which is outlined below. Sessions are administered to individual patients by the interventionist, who will be a trained research coordinator with adherence counseling experience (but who does not provide usual care). Each session lasts approximately 30 minutes. Exercises often involve completing worksheets or the use of handouts, which are given to the patient to use as a reference at home. Sessions are structured and manualized but still allow flexibility for tailoring the content of the exercises to the needs of the individual patient. Figure 1 depicts the flow of participation through each phase of the intervention.

##### Pretreatment training phase

This phase is composed of a series of up to four 1-week practice trials accompanied by adherence counseling and a dose regulation mechanism in which patients discontinue the practice trials and initiate ART once >85 % dose-taking adherence is achieved in a single practice trial, thus demonstrating adherence readiness. Using 85 % adherence to define readiness is consistent with what the literature indicates is needed for optimal treatment benefits with the new, more potent ART regimens [1–3]. With today’s common once-daily regimens, this threshold allows the patient to miss one dose over the course of the week and still meet the criteria for readiness. Early exercises and sessions will be focused on enhancing motivation and confidence to help prepare the patient for later skill-building exercises that may call for behavior change to overcome adherence barriers.

***Session 1*** Following an introduction and description of the ART regimen, the counselor will provide education about concepts such as viral load, drug resistance, and the importance of dose-timing to ensuring a constant adequate drug level. The importance of being ready to adhere well before starting treatment will be emphasized. Motivational interviewing (MI) techniques [16] will be used to help the patient develop or strengthen positive attitudes toward treatment and adherence, with the goal of improving adherence self-efficacy and motivation to adhere well. Exercises will be devoted to enhancing social support (reminders, transportation to clinic, provide reinforcement for successful adherence) provided by the patient’s social network. The practice trial will be introduced as an opportunity to experience what it is like to follow the prescribed ART regimen and to evaluate the patient’s readiness to start treatment. The practice trial regimen will mimic what the patient’s provider intends to prescribe. A 1-week supply of vitamins, with the electronic monitoring cap attached to one of the pill bottles, will be given to the patient to complete over the coming week.

***Sessions 2–5*** The number of sessions depends on the number of practice trials the patient needs to complete. Electronically monitored adherence results during the preceding week’s practice trial are reviewed with the patient. The electronic monitoring printout provides a chronology graph that depicts exactly the time that doses were taken each day. Good adherence is reinforced, and missed doses are identified, including any patterns that may be apparent. Patients are asked to identify barriers that contributed to missed or late doses, and antecedents to these scheduled doses are discussed. The stages of problem-solving are introduced (define the problem, decide on a goal, generate a list of possible solutions, compare and select a solution to try, plan the implementation of the solution, evaluate the effectiveness of the solution). MI techniques will be used, and the patient will be encouraged to take a leading role in identifying key barriers, coming up with potential solutions, and evaluating his or her experience in using these solutions, all of which are designed to maximize the patient’s feelings of autonomy and self-efficacy and increase the likelihood that these strategies will be successfully adopted.

***If dose-taking adherence during the practice trial is 85 % or greater*,** the patient will be considered ready to start ART, no further practice trials will be completed, and ART will be prescribed. The specific ART regimen planned for the patient will be reviewed. The patient will be asked to describe his or her daily routine and optimal ways for integrating the medication doses into his or her daily life. Doses will be connected with specific daily activities or behaviors so that these routinized activities can serve as reminder triggers for taking medication doses. Common side effects associated with the specific antiretrovirals prescribed will be discussed; side effect management handouts outlining possible strategies to manage specific side effects will be reviewed; and the patient will be encouraged to identify strategies that are feasible for to adopt if side effects occur.

***If dose-taking adherence to the practice trial is <85 %*,** the patient will be given another 1-week supply of vitamins for the next practice trial and encouraged to use the strategies identified in the session to overcome adherence barriers during the coming week. Linking the doses to routinized daily activities will be discussed. For those who are unable to achieve 85 % adherence after completing four practice trials, the decision whether to start treatment will be left up to the patient and his or her provider. Adherence results from the practice trials are shared with the patient’s provider to assist in making this decision. Similarly, adherence data once the patient is on ART, but none of the survey data, will also be shared with the provider.

##### Early treatment training phase

Training sessions are scheduled at weeks 2 and 4 following onset of ART to help patients maintain a high level of adherence readiness and self-efficacy once they start the actual ART regimen. There is no dose regulation during this phase, as all patients receive both sessions. Session content revolves primarily around identification of barriers, resolution of adherence barriers, and side effect management. The exceptions to this rule are the components of social support enhancement (week 2) and addressing attitudes toward treatment and adherence (week 4), which will take place in only one session each.

##### Maintenance training phase

The pre- and early-treatment phases help patients to be ready to adhere well at the beginning of treatment, but the skills needed to sustain adherence readiness over the long term may be quite different from those needed at initiation; hence the need for ongoing support. Maintenance modules will start at week 12 and thereafter at each routine clinic visit scheduled by the primary care provider as part of usual care, with the goal of helping patients sustain a high level of adherence over the course of treatment. Each module is composed of biweekly sessions and is dose-regulated such that patients who achieve >85 % dose-taking adherence during the prior month receive only one session while others receive sessions until >85 % adherence is achieved during the 2-week period preceding each successive session. For example, if the patient’s adherence from weeks 8 to 12 is 85 % or greater, then the patient will receive just the training session at week 12 and not return until the next routine clinic visit. However, if the patient’s adherence is <85 %, the patient will return for another session at week 14. The number of these biweekly sessions will be capped at three within each module. As with the pretreatment training, this dose regulation mechanism allows for varying the amount of training to the needs of the individual patient and for more efficient use of limited clinic resources for adherence support. The content of the maintenance training sessions will be similar to that during the early-treatment training phase, with continued emphasis on identification and resolution of adherence barriers, side effect management, and use of social support to help ensure commitment and motivation for long-term adherence.

##### Intervention training, supervision, and monitoring of fidelity

Fidelity in intervention administration will be achieved through training, supervision, and monitoring. The intervention counselors will be research coordinators experienced in coordinating HIV treatment research, including provision of adherence support during clinical trials. They are not involved in provision of usual care, thus limiting any risk of contamination of the usual care control. Training will include review of the manual, extensive role-playing of session exercises, and use of basic MI techniques. Sessions will be audiotaped and reviewed by the supervisor, who will use them to give feedback for improvement. The supervisor will listen to each session of the first three intervention participants of each counselor and then each session of every fifth participant of each counselor. Feedback will be provided and problem cases discussed during biweekly supervision.

### Measures

Below are descriptions of the primary and secondary outcomes with regard to ART adherence, virologic suppression, immune function and HIV care retention (see Table 2). The survey assessment includes measures of potential mediators of intervention effects (e.g., components of the IMB model such as HIV knowledge, adherence motivation and self-efficacy, as well as depression and substance use), and potential moderators (e.g., demographics), but these are not described in detail here; however, we do describe below our survey measures of adherence support received from HIV providers as part of usual care. The survey will be facilitated using computer-assisted self-interview technology, which automatically stores survey responses; survey data files will be stored on password-protected, encrypted files.

**Table 2:** Primary and secondary outcomes

Outcome measures	Outcomes
Primary outcomes	
HIV viral load (chart-abstracted HIV RNA)	Undetectable viral load (binary) (primary) Log change in viral load from baseline (continuous)
Dose-taking adherence (MEMS)	Took at least 85 % of prescribed doses (binary) (primary) Percentage of prescribed doses taken (continuous)
Secondary outcomes	
Dose-timing adherence (MEMS)	Percentage of prescribed doses taken in correct time window (±2 h for twice-daily regimens and ±3 h for once-daily regimens) (continuous) Took at least 85 % of prescribed doses on time (binary)
Dose-taking adherence (self-report)	Percentage of prescribed doses taken in past 7 days (continuous) Took at least 85 % of prescribed doses (binary) Visual analogue scale rating from 0 % to 100 % adherence over past 30 days
Dose interruptions (MEMS data)	Number of >48-h interruptions
Clinic attendance (chart abstract; self-report)	Number of attended and missed clinic appointments in past 6 months
CD4 count (chart-abstracted)	Change from baseline

*MEMS* Medication Event Monitoring System

#### ART adherence

Adherence to one of the vitamins in the practice trials for those in the intervention arm, and to one antiretroviral (with the most complex regimen) for all participants once ART is initiated, will be measured electronically and continuously throughout the study using the Medication Event Monitoring System (MEMS) caps. MEMS caps house a microelectronic chip that records the date and time of each bottle-opening, enabling a precise, objective assessment of the timing of each dose and the patient’s pattern of pill-taking. Participants will be instructed to refill the bottle when removing the last remaining dose, to use the bottle provided for the monitored medication, and to remove only one dose at a time when the medication will be ingested. Participants do not always follow these instructions (e.g., removing multiple doses at once or “pocketing,” or opening the bottle without removing a dose), so we will assess this via self-report using a “past 2 weeks” time frame at each primary assessment and will adjust the number of prescribed does taken during this time period, which has been validated and shown to strengthen the relationship between adherence and viral load [17]. We will ask patients to notify the coordinator if their provider changes their ART regimen.

Data taken from the caps are downloaded into a software file that calculates summary scores that include percentages of prescribed doses taken (dose-taking adherence) and prescribed doses taken within a specified window of time (dose-timing adherence). For the analysis, we will examine both mean dose-taking adherence and proportion with ≥85 % dose-taking adherence as primary outcomes, as well as mean dose-timing adherence and proportion ≥85 % dose-timing adherence as secondary outcomes. We will also ask participants to self-report the number of doses missed in the last week and percentage of prescribed medications taken in the last month, which will be used as secondary outcomes.

#### HIV care retention

At each survey assessment, clients are asked about their number of HIV care provider appointments scheduled, attended, and missed in the past 6 months.

#### Virologic suppression and immune status

Clients will be asked to provide informed consent for access to medical record data, which we will use to access HIV viral load and CD4 assay results from the clinic’s electronic medical charts. These assays are performed routinely as part of usual care at regular clinic visits, which are scheduled every 3–6 months for patients on ART. Undetectable viral load and change in log-transformed mean viral load are primary outcomes; change in CD4 count is a secondary outcome.

#### Assessment of usual care practices to enhance adherence

Participants will be asked at each follow-up assessment whether a provider has engaged them in specific adherence-related discussions. Participants will be asked to “indicate whether or not your provider(s) discussed the following with you to help you adhere to your ART regimen during the last 6 months.” For example, “Did your providers discuss with you how to reduce or overcome any problems you’ve had in adhering to your ART regimen?” Participants respond to each of six items using a 3-point response scale, with 0 = “No, this was never discussed”; 1 = “Yes, a little time was spent discussing this”; and 2 = “Yes, a lot of time was spent discussing this.”

### Data safeguarding and monitoring plan

Only U.S. Food and Drug Administration–approved antiretroviral medications prescribed and monitored by the primary care providers as part of routine clinical care will be used in the study. Thus, we do not anticipate any medication-related adverse events beyond those seen in routine HIV medical care. Any adverse medication events that occur as part of usual medical care and noted by study staff will be communicated with the participant’s consent to their primary medical care provider. Loss of confidentiality is a potential adverse event, and we have set up several mechanisms to ensure confidentiality of participants. The nature of any information that may need to be disclosed to protect the participant or others is included as part of the consent process.

A single independent monitor will be appointed to oversee the study. To allow effective monitoring, the independent monitor will be provided with periodic reports, which will include subject enrollment, subject retention, the number of patients who drop out of the study with reasons for dropping out, and a listing of all adverse events that are plausibly related to study procedures. Periodic reports will be provided to the independent monitor at 6-month intervals; however, adverse events that are considered directly related to aspects of study participation will be reported immediately to the monitor, the IRB, and the NIH. After review of the periodic reports, the independent monitor may ask for clarification or additional information from the principal investigator. After such information is provided, if requested, the independent monitor will make a recommendation regarding the continuation, modification, or termination of the study. All communications from the independent monitor will be shared with the IRBs and the NIH.

We have set up several mechanisms to ensure the confidentiality of data collected from the participants. All paper files are stored in locked file cabinets, and electronic files are stored in password-protected, encrypted files. Furthermore, both paper and electronic files are identified only by a participant’s identification number. Identifying information linking participants to their study identification number is retained in a locked cabinet accessible only by the principal investigator and study coordinator. The final dataset will reside with the principal investigator.

### Data analysis

The primary aim of the analysis is to determine whether the START intervention is superior to usual care in helping patients achieve optimal adherence and virologic suppression in both the short and long term. Dose-taking adherence and viral load are the primary outcomes. Continuous adherence will be assessed for normality and transformed if needed; viral load data will be log-transformed. Our primary analysis is based on an intent-to-treat approach, with secondary analyses involving study completers. Attrition weights will be used to account for dropouts, and all analyses will incorporate design effects from this weighting in the calculation of standard errors and tests of significance. Analyses will be conducted to compare groups at months 6 and 24 to assess short- and long-term effects.

In addition to simple between-group comparisons at each time interval (baseline and months 6, 12, 18, and 24), we will conduct analysis of the trajectory of outcomes. Longitudinal analyses will be performed, using mixed effects models to examine differences in trajectories of adherence and HIV viral load across the two groups, controlling for patient characteristics. Linear mixed effects models will be used for continuous outcomes, while nonlinear mixed effects models will be used for binary outcomes (e.g., took at least 85 % of prescribed doses, undetectable viral load). Along with a fixed linear term for time, a random subject-specific intercept will be incorporated, with the appropriate covariance structure of the repeated measures being determined during analysis. Covariates added to the models will include components of our conceptual IMB framework and intervention (e.g., HIV knowledge, adherence motivation, adherence self-efficacy), as well as correlated process variables (e.g., number of practice trials completed, total number of sessions attended). To adequately model longitudinal HIV viral load, which has a high proportion of values below the limit of detection, a Bernoulli/log-normal random effects mixture model with left censoring will be used [18, 19]. Analysis of covariance will be used to define an effect size estimate in the analysis of the primary outcomes and to provide baseline adjustment to improve statistical power.

A fully specified statistical analysis plan will be written before unmasking of the data. The article presenting the primary findings from this study will follow the outline provided in Additional file 1. Study findings will be disseminated to researchers and clinicians via publications and conference presentations and to study participants via a mailed one-page summary of findings. Authorship of published papers will follow established guidelines for defining the level of contribution that warrants authorship. Public access to study data will be made available upon request and review of registration of application.

## Discussion

In this study, we will conduct a rigorous, controlled evaluation of the innovative ART adherence intervention START. Unlike most other ART adherence interventions that target people who are already on ART [12, 15, 20], START attempts to provide the field with an evidence-based intervention that works with patients before they start treatment to ensure adherence readiness before initiation—an approach that is particularly timely as more patients with early stage disease start ART and may be less ready to adhere well.

An added innovation of START is highlighted in its design, which features components that are lacking in most ART adherence interventions to date: (1) inclusion of pretreatment, early-treatment, and ongoing maintenance training phases; (2) use of brief practice trials for determining adherence readiness and the timing of ART initiation; and (3) inclusion of a dose regulation mechanism that adjusts the amount of pretreatment and maintenance training based on the patient’s recent adherence performance. START combines these features with a goal of producing a robust and lasting effect on both adherence and clinical outcomes, as well as to be sensitive to and consistent with community practice, the limited resources available to most clinics and providers (especially as more patients start ART), and the wide range of adherence skills present among patients. This flexibility and attention to individual patient needs should increase the acceptability of the program to patients, providers, and clinic administrators, thus improving the likelihood of future translation into usual care.

Our pilot controlled trial of START revealed evidence of the intervention’s potential promise, with medium to large effects on both behavioral adherence and undetectable viral load [14]. If this larger, fully powered controlled test of the intervention produces similar results, START will provide clinics with a model for a “new-generation” adherence support program to assist their patients in achieving optimal treatment outcomes. Also, this may be the first study of an adherence intervention with a 2-year intervention and follow-up period, which will enable us to assess the intervention’s long-term efficacy.

## Trial status

Enrollment is active.

## Additional file

Additional file 1: 2010 Consolidated Standards of Reporting Trials (CONSORT) flow diagram of the trial. (DOC 46 kb)

## Abbreviations

ART: antiretroviral therapy
CARE: California AIDS Research and Education Center
IMB: information-motivation-behavioral skills model
IRB: institutional review board
MEMS: Medication Event Monitoring System
MI: motivational interviewing
NIH: National Institutes of Health
RZ: randomized
START: Supporting Treatment Adherence Readiness through Training
UCLA: University of California, Los Angeles
