# Supplementary material for: Supporting Treatment Adherence Readiness through Training (START) for patients with HIV on antiretroviral therapy: study protocol for a randomized controlled trial
Source: Trials. 2016 Mar 24;17:162. doi: 10.1186/s13063-016-1287-3 (PMC4806419; doi:10.1186/s13063-016-1287-3)
Supplement: Additional file 1: — 2010 Consolidated Standards of Reporting Trials (CONSORT) flow diagram of the trial. (DOC 46 kb) [file 13063_2016_1287_MOESM1_ESM.doc]

**
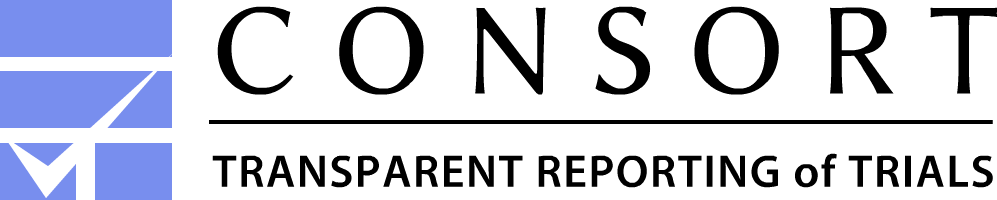
**

**CONSORT 2010 Flow Diagram**

**Allocation**

**Analysis**

**Follow-Up**

**Enrollment**

Assessed for eligibility

Primary analysis uses intention to treat approach, so all randomized participants are included

Follow-up surveys conducted at months 6, 12, 18, and 24

Allocated to START intervention

Follow-up surveys conducted at months 6, 12, 18, and 24

Allocated to usual care control

Primary analysis uses intention to treat approach, so all randomized participants are included

Randomization
